# Encouraging impact of doxycycline on early mortality in cardiac light chain (AL) amyloidosis

**DOI:** 10.1038/bcj.2017.26

**Published:** 2017-03-24

**Authors:** A D Wechalekar, C Whelan

**Affiliations:** 1 National Amyloidosis Centre, University College London (Royal Free Campus), London, UK

Early mortality in cardiac light chain (AL) amyloidosis has not changed substantially over the last 25 years, although long term outcomes have improved.^1^ In addition to the physical effects of AL fibril deposition causing cardiac dysfunction, amyloidogenic light chains are cardiotoxic, reducing contractility in cardiac myocytes,^2^ causing early death in zebra fish^3^ and reducing pharyngeal contractility in *C.elegans*.^4^ Blocking light chain cardiotoxicity by small molecules is attractive. Doxycycline interferes with amyloid fibril formation in a mouse model of AL amyloidosis^5^ and abrogates light chain toxicity *in vitro*.^4^ Doxycycline combined with tauroursodeoxycholic acid improved cardiac outcomes in transthyretin amyloidosis. In a cohort of patients with systemic AL amyloidosis, doxycycline given as antimicrobial prophylaxis post autologous stem cell transplantation significantly improved survival compared to controls who were given penicillin.^6^ We and others reported that treatment with highly effective bortezomib-based chemotherapy does not improve early survival in AL amyloidosis.^7, 8^ Hence, an agent that directly interferes with the amyloidogenic pathway targeting either misfolded light chains or the amyloid fibrils is needed.

We report retrospective outcomes of adding doxycycline to standard chemotherapy in 30 patients with cardiac AL amyloidosis compared to 73 matched controls (matched for cardiac disease stage,^9^ absolute N-terminal brain natriuretic peptide (NT-proBNP) level, age and free light chains (dFLC)). Ninety-four percent had Mayo stage III disease (28 were Mayo stage IIIb). Table 1 shows the baseline characteristics in both groups. An additional analysis of patients treated with only with bortezomib-based regimes in both arms was done. The median NT-proBNP was 4728 pmol/l (range, 559–37 889 ng/l); high sensitivity troponin 0.1 μg/l (range, 0.032–0.95 μg/l) and dFLC 505 mg/l (range 54–3428), with no significant difference between either group. Doxycycline was given 100 mg twice daily after meals. The median duration of doxycycline was 6 months (range, 1–24 months). Initial treatment regimens were: bortezomib, thalidomide and alkylators based in 72%, 23% and 5%, respectively. Three patients discontinued doxycycline due to toxicity (Grade 2 photosensitive rash—2; grade 3 nausea/vomiting—1). Responses to treatment were analysed on an intent-to-treat basis (ITT), patients who died before response assessment were classified as non-responders. The haematologic response on an ITT at the end of cycle 1 of chemotherapy was not significantly different in the group (doxycycline vs controls: very good partial response (VGPR) or better—31% vs 29% partial response (PR)—31% vs 35% no response (NR)—38% vs 32%). The overall response rate (PR or better) on an ITT basis was significantly better in the doxycycline vs control groups (93% vs 59% *P*=0.001) with: complete response—56% vs 35% (*P*=0.001), VGPR—10% vs 8% (*P*=0.32) and PR 30% vs 35% (*P*=0.66), respectively (Figure 1a). On an ITT basis, the median overall survival was 13 months in the control group and not reached in the doxycycline group. The 12 and 24 month survival in the doxycycline vs control group was 82% vs 53% and 82% vs 40%, respectively (*P*<0.0001), with marked improvement on OS for patients with Mayo stage IIIa (Figure 1b) and high presenting troponin T (>0.1 μg/l). On an ITT, 60% of the doxycycline group, and 18% of the control group achieved cardiac responses (*P*<0.0001) (Figure 1c). Use of doxycycline remained an independent predictor of better outcomes in ITT and 6 month landmark analysis. When analysis was restricted to patients with a haematologic and/or a cardiac response (80% of total in doxycycline group vs 42% of total in the control group), at 12 and 18 months in control and doxycycline groups respectively, 75% vs 88% and 65% vs 88% were alive (log rank *P* =0.24).

A total of 76 patients (26 in the doxycycline group and 50 in the control group) were treated with upfront bortezomib-based regime. Among the bortezomib-treated patients, there was a non-significantly greater number of haematologic responses in the doxycycline-treated group vs controls (61% vs 46% (*P*=0.23)). However, there was still a significant improvement in the median survival of the doxycycline-treated group, the median survival in the control group was 15 months vs not reached in the doxycycline group (log rank *P*=0.012; 58% vs 84% survival at 12 months respectively) (Figure 1d). In the bortezomib-treated subgroups, there is significantly greater decrease in NT-proBNP in the doxycycline group compared to controls (mean decrease 5700 ng/l vs 1127 ng/l; *P*=0.035).

In conclusion, this study suggests that addition of doxycycline to standard chemotherapy reduces mortality in cardiac AL amyloidosis. The particular impact on patients presenting with a high troponin in particular and significantly greater decrease in NT-proBNP suggests that doxycycline may reduce of light chain cardiotoxicity as one of the possible mechanisms of action. The benefit of doxycycline appeared greatest in patients with Mayo stage IIIa disease and limited, if any, in the very advanced stage IIIb patients. There was lack of significant difference in the clonal response rates between the doxycycline vs control group, at end of one month and (for evaluable patients only) at six months. Moreover, for the patients who survived and achieved a haematologic response, there was only a non-significant improvement in survival in the doxycycline group at 12 and 18 months compared to controls. This suggests that firstly, there is no direct impact of doxycycline supplementation on the actual efficacy chemotherapy and critically, the main impact of doxycycline is reduction in early cardiac mortality. It is reduction in the early deaths in the doxycycline-treated group that allows greater proportion of patients to live long enough to achieve deeper clonal responses to chemotherapy and consequent cardiac responses. These data need to be interpreted with caution due to limitations of small sample size and reporting bias of small early studies. Additionally, the exact reason for the reduction in early mortality remains unclear—whether this is due the reduction of the light chain toxicity, impact on other aspects of cardiac dysfunction independent of its effect on light chains/amyloid or indeed its antibiotic properties. Other therapies like novel monoclonal antibodies (NEO001D^10^ and GSK2398852^11^) which accelerate clearance of amyloid deposits by activating tissue macrophages are in advanced phase clinical trials. Since the mechanism of action of doxycycline is likely to be different, if the activity is confirmed, it may offer synergy with therapies that clear amyloid deposits. These encouraging early results need formal confirmation in a prospective randomised controlled trial before this can be adopted in routine clinical practice.

## Figures and Tables

**Figure 1:**
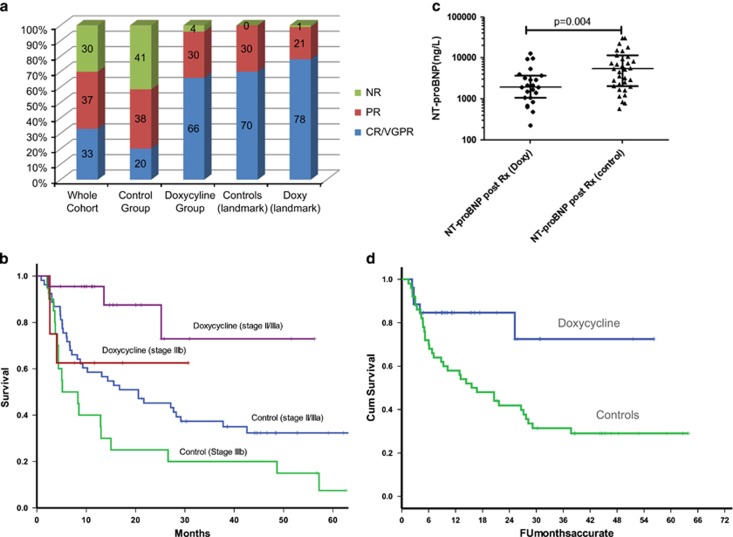
(**a**) The percentage of haematologic responders in the doxycycline and control groups. On an ITT, the overall response rate was significantly better in the doxycycline-treated group (the patients who died before response assessment are counted as non-responders). However, in the landmark cohort, there is no significant different in the haematologic responses confirming that doxycycline *per se* does not influence the response to chemotherapy, but only does so by allowing more patients in the treated group to live longer and achieve a response to chemotherapy. (**b**) Overall survival in the doxycycline-treated and control cohorts divided by the Mayo disease stage. Patients with very advanced cardiac stage IIIb disease (defined as NT-proBNP >8500 ng/l) do not have significant improvement in survival but patients with stage II/IIIa (NT-proBNP <8500 ng/l) have a significant improvement in overall survival when treated with doxycycline compared to matched controls. (**c**) Significantly greater reduction in NT-proBNP after completion of treatment in the doxycycline-treated group compared to the controls. (**d**) Survival of patients treated with an upfront bortezomib-based regime stratified by doxycycline vs controls. The median survival was 15 months in the control group and was not reached in the doxycycline group (log rank *P*=0.012).

**Table 1:** Baseline characteristics

*Clinical characteristic*	*All*	*Doxycycline group*	*Control group*	P*-value*
Age (years)	65 (41–84)	63 (41–85)	67 (41–87)	0.54
Sex (male, %)	69 (67%)	21 (70%)	48 (65%)	0.49
ECOG>1 (%)	68 (67%)	26 (87%)	43 (56%)	
				
*Monoclonal protein type (%)*				
IgG	34 (33%)	7 (33%)	27 (37%)	
IgA	12 (11%)	–	12 (16%)	
IgM	1 (1%)	–	1 (2%)	
Light chain only	34 (33%)	15 (50%)	19 (26%)	
Lambda (%)	72 (69%)	22 (73%)	64 (87%)	
Median dFLC (mg/l)	303 (43–3245)	367 (62–3245)	262 (62–3428)	0.34
				
*Organ involvement (%)*				
Renal	63 (61%)	15 (50%)	48 (65%)	0.18
Cardiac	103 (100%)	30 (100%)	73 (100%)	
Hepatic	14 (13%)	4 (13%)	10 (14%)	0.08
Peripheral nerves	7 (6%)	3 (10%)	4 (6%)	0.2
Autonomic nerves	10 (9%)	4 (13%)	6 (8%)	0.2
Gastrointestinal	8 (7%)			
Soft tissue	18 (17%)	3 (10%)	15 (20%)	0.14
Other	2 (2%)	1 (3%)	1 (2%)	0.23
⩾2 organs involved (%)	74%	73%	56 (78%)	
Median creatinine	101 (40–483)	99 (49–483)	102 (40–269)	0.82
Median eGFR	63 (15–100)	65 (15–100)	59 (22–100)	0.44
				
eGFR<45 ml/min (%)				
Median 24 h urine protein	1.55 (0–22)	0.25 (0.1–12)	2.2 (0.1–22)	0.54
Albumin	36 (15–48)	40 (18–47)	32 (15–48)	0.001
Median alkaline phosphatase	85 (35–1028)	84 (35–593)	86(35–1028)	0.89
				
Median ventricular wall thickness (mm)	14.5 (13–21)	15 (12–19)	14 (12–21)	0.33
Median ejection fraction, %	55 (29–70)	55 (32–70)	55 (29–70)	0.64
Median NT-proBNP (ng/l)	4728 (559–37889)	4317 (1211–33872)	4940 (559–37889)	0.98
NT-proBNP>8500 (%)	34 (33%)	8 (26%)	26 (35%)	0.49
Median HS-Troponin T	0.1 (0.003–0.95)	0.1 (0.013–0.65)	0.1 (0.003–0.95)	0.70
Median systolic BP	113 (84–157)	115 (85–144)	111 (84–157)	0.60
				
*Mayo cardiac stage (%)*				
I	0	0	0	
II	10 (10%)	5 (16%)	5 (7%)	
III	93 (90%)	25 (83%)	68 (93%)	
IIIb	34 (33%)	8 (26%)	20 (27%)	
